# Heart rate variability and its relationship with central and general obesity in obese normotensive adolescents

**DOI:** 10.1590/S1679-45082013000300005

**Published:** 2013

**Authors:** Breno Quintella Farah, Wagner Luiz do Prado, Thiago Ricardo dos Santos Tenório, Raphael Mendes Ritti-Dias

**Affiliations:** 1 Universidade de Pernambuco, Recife, PE, Brazil; Graduate Associate Program on Physical Education,; Universidade Federal da Paraíba

**Keywords:** Obesity, Anthropometry, Heart rate, Autonomic nervous system, Adolescent

## Abstract

**Objective:**

To analyze the relationship between the heart rate variability parameters and the indicators of central and general obesity in obese normotensive adolescents.

**Methods:**

Seventy-four 13 to 18 year-old obese normotensive adolescents participated in this study. The indicators analyzed for central and general obesity were waist circumference and body mass index, respectively. Heart rate variability was obtained by heart rate monitoring. For this, the adolescents remained in a supine position for 7 minutes with controlled breathing. Parameters were obtained in time domain (standard deviation of all the RR intervals, root mean square of successive differences between the normal adjacent RR intervals and the percentage of adjacent intervals with more than 50ms) and frequency domain variables (low and high frequency bands and the sympathovagal balance).

**Results:**

After adjustments for gender, age, and cardiorespiratory fitness, a negative correlation between the waist circumference and the root mean square of successive differences between the normal adjacent RR intervals (ß=-1.51; standard error=0.56; p<0.05) and the percentage of adjacent intervals with more than 50 ms (ß=-0.96; standard error=0.34; p<0.05) were observed, while the body mass index showed no significant correlation with any heart rate variability parameter (p>0.05).

**Conclusion:**

Central obesity is a better discriminator than general obesity of autonomic cardiac dysfunction in obese normotensive adolescents

## RESUMO

**Objetivo:**

Analisar a relação entre os parâmetros da variabilidade da frequência cardíaca e os indicadores de obesidade central e geral em adolescentes obesos normotensos.

**Métodos:**

Participaram deste estudo 74 adolescentes obesos normotensos, com idade entre 13 e 18 anos. Os indicadores de obesidade central e geral analisados foram a circunferência da cintura e o índice de massa corporal, respectivamente. A variabilidade da frequência cardíaca foi obtida por meio de monitor de frequência cardíaca. Para tanto, os adolescentes permaneceram na posição supina durante 7 minutos e com respiração controlada. Foram calculadas as variáveis do domínio do tempo (desvio padrão de todos os intervalos RR, a raiz quadrada da média do quadrado das diferenças entre os intervalos RR normais adjacentes e a percentagem dos intervalos adjacentes com mais de 50ms) e do domínio da frequência (bandas de baixa e alta frequência e o balanço simpatovagal).

**Resultados:**

Após ajustes para gênero, idade e capacidade cardiorrespiratória, foi observada relação negativa entre a circunferência da cintura e a raiz quadrada da média do quadrado das diferenças entre os intervalos RR normais adjacentes (ß=-1,51; erro padrão=0,56; p<0,05) e a percentagem dos intervalos adjacentes com mais de 50ms (ß=-0,96; erro padrão=0,34; p<0,05), enquanto o índice de massa corporal não teve relação significante com nenhum parâmetro da variabilidade da frequência cardíaca (p>0,05).

**Conclusão:**

A obesidade central é melhor discriminadora de disfunção autonômica cardíaca em adolescentes obesos normotensos do que a obesidade geral.

## INTRODUCTION

Over the last years, the prevalence of obesity has grown rapidly among children and adolescents^(1)^. During the initial phases of life, obesity has been linked to the early onset of chronic diseases, such as type 2 diabetes and cardiovascular diseases^(2,3)^. The risk of developing hypertension, a known risk factor for cardiovascular diseases, is greater in obese adolescents than in eutrophic adolescents^(4)^.

Heart rate variability (HRV), a method that consists of the analysis of different parameters based on the time variation between successive heart beats, has been used to quantify cardiac parasympathetic and sympathetic autonomic modulation^(5)^.

HRV may be analyzed by linear methods, which are divided into two domains: time and frequency. The time domain is based on the variations of cardiac cycles considered normal (RR interval) within a given time, and its parameters are obtained from statistical methods in the RR intervals such as mean, measures of dispersion, and count. On the other hand, the frequency domain uses the quantification of spectral density of potency by means of specific mathematical algorithms to decompose HRV into oscillatory components with defined frequencies^(5)^.

In a healthy autonomic nervous system, it is expected that at rest there is a predominance of parasympathetic cardiac modulation. Conversely, in individuals with heart disease, there is greater sympathetic modulation and lesser parasympathetic modulation of the heart^(6)^. Thus, HRV emerges as an important indicator of regulation alterations in the cardiovascular system and can provide information on the behavior of the cardiac autonomic system in different populations^(5,6)^.

Hypertensive individuals present HRV alterations, a cardiac autonomic balance in favor of sympathetic modulation^(7,8)^. These changes have been attributed to the effects of obesity on the autonomic nervous system, since many hypertensive individuals are also obese^(9–12)^. Nevertheless, the relationship between obesity and autonomic dysfunction seems to depend on the indicator of obesity used^(13–15)^. For example, Chen et al.^(13)^, in analyzing 28 obese individuals with a mean age of 29 years, observed that central obesity indicators (waist circumference and waist/hip ratio) were related to alterations in autonomic modulation, while the general obesity indicator (body mass index) showed no significant relationship.

In adolescents, data on this theme are scarce. Guizar et al.^(11)^, when analyzing 70 adolescents aged between 12 and 17 years, verified that both central and general obesity are related to alterations in autonomic modulation. However, only boys were analyzed, which limits the extrapolation of results for the girls, considering that there is a difference between genders in cardiac autonomic modulation^(16)^. Additionally, other factors that influence cardiac autonomic modulation, such as cardiorespiratory capacity, were not considered in the analyses. In fact, children and adolescents with greater cardiorespiratory capacity have greater parasympathetic modulation and lesser sympathetic modulation at rest when compared to their less fit peers^(17,18)^. Therefore, this fact needs to be considered as well.

## OBJECTIVE

To analyze the relationship between the parameters of heart rate variability and indicators of central and general obesity in obese normotensive adolescents.

## METHODS

In this cross-sectional study, 74 obese adolescents (47 girls and 27 boys) were included from three cohorts (2010, 2011, and 2012) of the multidisciplinary program for the treatment of obesity in adolescents at the *Universidade de Pernambuco*. The adolescents were recruited by means of television, radio, and local newspaper announcements. Inclusion criteria were body mass index greater than or equal to the 95^th^ percentile for age and gender^(19)^; age between 13 and 18 years; Tanner maturation stage between 3 and 4^(20)^; normal blood glucose level (<126 mg/dL)^(21)^; and normal blood pressure (blood pressure lower than the 95^th^ percentile for age, height, and gender)^(22)^.

The study was approved by the Research Ethics Committee of the *Universidade de Pernambuco* (154/09). All parents or legal guardians signed the Informed Consent Form for voluntary participation in the study.

### Indicators of obesity

The adolescents were weighed with light clothing and no shoes on automatic scales with 0.1kg precision (Filizola model 160/300, Brás, Brazil). Height was measured by a wooden stadiometer with 0.01m precision. Body mass index was calculated by the coefficient between body mass and height squared (kg/m^2^). Waist circumference was measured by the smallest circumference between the last rib and the iliac crest. The body mass index and the waist circumference were considered indicators of general and central obesity, respectively.

### Evaluation of blood pressure

Blood pressure was measured after 5 minutes of resting in sitting position, using a mercury column sphygmomanometer Missouri™ and appropriate cuffs sizes. Readings of the first and fifth Korotkoff phases were adopted as systolic and diastolic blood pressures, respectively. Triplicate measurements were made for each arm, using the arm with the highest pressure for analyses^(22)^.

### Evaluation of cardiorespiratory capacity

Oxygen uptake was measured directly by a continuous incremental protocol using a treadmill (Cosmed T200, Rome, Italy), as previously described^(23)^. Treadmill grade was set at 1% and the initial speed was maintained at 4km/h during the first 3 minutes. After this period, speed was increased 1km/h a minute. Criteria for the test interruption were volitional fatigue, subjective perception of effort greater than 18 on the Borg scale^(24)^, and gas exchange rate greater than 1.15. The largest volume of oxygen (VO_2_) obtained before interruption of the test was considered the VO_2peak_. VO_2_ and the production of carbon dioxide (VCO_2_) were shown every 15 seconds, using an open circuit of the respiratory metabolic system (Quark PFT, Cosmed, Rome, Italy).

### Analysis of heart rate variability

Before HRV collection, all adolescents were instructed to maintain their normal sleep pattern, not ingest beverages with caffeine or alcohol, and not perform physical exercise 24 hours before the evaluations.

For the HRV analysis, the adolescents remained in the supine position for a period of 7 minutes, a time in which the RR intervals were obtained by means of a heart rate monitor (Polar model RS800CX, Polar Electro Oy Inc., Kempele, Finland), with breathing controlled (one respiratory cycle every 8 seconds) by a metrometer. Thus, the parameters of time domain, such as mean of the RR intervals, standard deviation of all normal RR intervals (SDNN), root mean square successive difference between normal adjacent RR intervals (RMSSD), and the percentage of adjacent intervals with more than 50ms (PNN50) were determined^(5)^.

**Figure 1:**
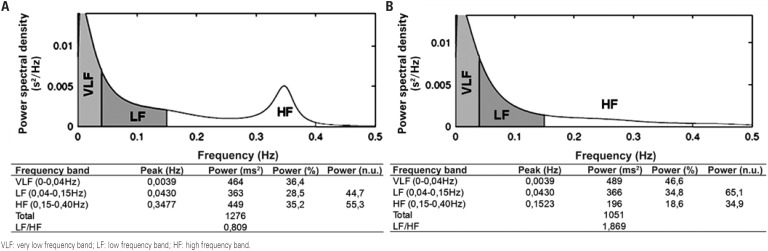
Spectral analysis of frequency using auto regression model, with order model 12 as per Akaike criterion for obese adolescents. Panel A: signal with predominance of parasympathetic modulation; panel B: signal with predominance of sympathetic modulation

The parameters of the frequency domain were determined by the HRV spectral analysis technique. Stationary periods of the tachogram for at least 5 minutes were decomposed into low and high frequency (LF and HF, respectively) bands by the autoregressive method, with order model 12 as per Akaike criterion. Frequencies between 0.04 and 0.4Hz were considered physiologically significant; the LF was represented by oscillations between 0.04 and 0.15Hz, and the HF between 0.15 and 0.4Hz. The power of each spectral component was calculated in normalized terms (nu). Normalization was done by dividing the power of each band by the total power, from which the value of the very low frequency band (<0.04Hz) was subtracted, and the result was multiplied by 100^(5)^. Examples of analysis of the frequency domain for obese adolescents may be seen on figure 1.

All HRV analyses were made by means of the Kubios HRV Analysis Software 2,0 for Windows (The Biomedical Signal and Medical Imaging Analysis Group, Department of Applied Physics, University of Kuopio, Finland) program and were performed by an experienced researcher that was blind as to the other variables.

### Statistical analysis

Bivariate and multiple linear regression analyses were conducted to verify the relationship between the HRV parameters and the indicators of obesity (body mass index and waist circumference). On the bivariate analysis, the relationship between the HRV parameters and the body mass index or the waist circumference was tested in separate regressions and with no adjustment for any variable (raw analysis). In the multiple analysis, the relationship between the HRV parameters and body mass index or waist circumference were adjusted for the variables of theoretical confounding, namely gender^(16)^, age^(25)^, and VO_2peak_^(17,18)^. These variables were maintained in the model, regardless of their significance.

The analysis of the residue was carried out and, for each model, the supposition of homoscedasticity and adherence to normal distribution was followed.

All statistical procedures were performed with the Statistical Package for the Social Sciences (SPSS), version 20.0. A value of p<0.05 was considered significant, and the data were presented as mean and standard deviation.

## RESULTS

The clinical characteristics and the HRV parameters are shown on tables 1 and 2. The mean age of adolescents was 16 years and the values of blood pressure were in normal range. Mean of the RR intervals was 790±116ms, SDNN was 79±31ms, RMSSD was 59±31ms, and PNN50 was 29±19. Additionally, it was noted that the adolescents presented a sympathovagal balance in favor of sympathetic modulation, since the mean LF/HF was 3.4±1.7, the LF band was 74±10nu, and the HF band was 26±10nu.

**Table 1:** Clinical characteristics of the adolescents (n=74)

Variables	Mean±standard deviation
Age (years)	15.9±1.5
Height (cm)	163.9±8.0
Body mass (kg)	93.0±12.5
Body mass index (kg/m^2^)	34.6±4.1
Waist circumference (cm)	96.4±7.9
Systolic arterial pressure (mmHg)	115±9
Diastolic arterial pressure (mmHg)	73±8
VO_2peak_ (ml.kg^-1^min^-1^)	22.3±5.9

**Table 2:** Heart rate variability parameters in obese normotensive adolescents (n=74)

Variables	Mean±standard deviation	Amplitude
Mean of RR intervals (ms)	790±116	576-1.079
SDNN (ms)	79±31	26-179
RMSSD (ms)	59±31	11-136
PNN50 (%)	29±19	0-69
LF/HF	3.4±1.7	0.6-9.1
LF (nu)	74±10	38-90
HF (nu)	26±10	10-62

SDNN: standard deviation of all normal RR intervals; RMSSD: root mean square successive difference between normal adjacent RR intervals; PNN50: percentage of adjacent intervals with more than 50 ms; LF/HF: sympathovagal balance; LF: low frequency band; HF: high frequency band.

Table 3 shows the multiple linear regression analyses. After adjustment for gender, age, and VO_2peak_, a statistically significant correlation was noted between waist circumference and RMSSD (r^2^=0.15; F=2.69; p=0.039) and PNN50 (r^2^=0.16; F=2.81; p=0.033), whereas the body mass index had no significant correlation with any HRV parameter (p>0.05).

**Table 3:** Relationship between indicators of obesity and heart rate variability parameters in normotensive obese adolescents

HRV parameters		WC β (SE)	BMI β (SE)
RR intervals (ms)	Raw	-3.72 (1.66)*	-5.09 (3.23)
	Adjusted**	−4.91 (2.18)	−5.50 (4.17)
SDNN (ms)	Raw	−1.01 (0.45)*	−0.52 (0.88)
	Adjusted**	−1.25 (0.56)	−0.77 (1.08)
RMSSD (ms)	Raw	−1.14 (0.43)*	−0.87 (0.86)
	Adjusted**	−1.57 (0.56)*	−1.38 (1.09)
PNN50 (%)	Raw	−0.75 (0.26)*	−0.63 (0.52)
	Adjusted**	−0.96 (0.34)*	0.69 (0.67)
LF/HF	Raw	0.02 (0.02)	0.02 (0.05)
	Adjusted**	−0.01 (0.03)	−0.05 (0.06)
LF (nu)	Raw	0.07 (0.15)	−0.01 (0.29)
	Adjusted**	−0.14 (0.21)	−0.42 (0.39)
HF (nu)	Raw	−0.07 (0.15)	0.01 (0.29)
	Adjusted**	0.14 (0.21)	0.42 (0.39)

* p<0.05;

** Adjusted by gender, age, and VO_2peak_.

WC: waist circumference; β: coefficient of regression; SE: standard error; BMI: body mass index; SDNN: standard deviation of all normal RR intervals; RMSSD: root mean square successive difference between normal adjacent RR intervals; PNN50: percentage of adjacent intervals with more than 50ms; LF/HF: sympathovagal balance; LF: low frequency band; HF: high frequency band; HRV: heart rate variability.

## DISCUSSION

The present results demonstrate that the indicator of central obesity had a negative correlation with RMSSD and PNN50, suggesting that a larger waist circumference is related to a smaller cardiac parasympathetic modulation in obese normotensive adolescents. On the other hand, the indicator of general obesity, measured by the body mass index, had no significant relationship with any HRV parameter.

Adolescents who are obese present greater cardiac sympathetic modulation and lesser cardiac parasympathetic modulation compared to adolescents of normal weight^(9–12)^. Nonetheless, the influence of the distribution of adipose tissue on cardiac autonomic modulation of adolescents still requires further investigation.

The results of the present study demonstrated that the largest waist circumference was related to the smallest parasympathetic modulation, and consequently, a greater cardiac autonomic dysfunction. Similar results were found in adults and elderly, which suggests that the indicators of central obesity are more sensitive than the indicator of general obesity, especially among obese^(13–15)^. On the other hand, Guizar et al.^(11)^ observed that both central and general central obesity were related to the sympathovagal balance of the heart in favor of sympathetic modulation in adolescents. Nevertheless, the relationship of the indicators of obesity with cardiac autonomic modulation was performed with normal weight and obese adolescents, which could explain the differences between our and this study.

It is speculated^(17,18)^ that cardiorespiratory capacity has a positive relationship with parasympathetic cardiac modulation and that, with an increase in age, there is a decrease of parasympathetic modulation^(25)^. Additionally, it is known that girls show greater parasympathetic modulation than boys^(16)^. Therefore, in the present study, the statistical analyses were adjusted by VO_2peak_, principal indicator of cardiorespiratory capacity by gender and by age. Interestingly, the relationship between waist circumference and the HRV parameters remained significant after adjustment for these variables, indicating that central obesity, compared to general obesity, is the best discriminator of autonomic cardiac dysfunction in obese adolescents, even after adjustment for the intervenient variables.

Although the mechanisms of these responses have not yet been evaluated, it is known that fat cells are responsible for secreting various adipokines, among them leptin, which is responsible for activating the neural pathways that increase the activity of the sympathetic nervous system^(26,27)^. Although the metabolic activity of the central adipose tissue might explain the relationship with the smaller parasympathetic modulation, the lack of a significant relationship between autonomic modulation and body mass index is still not clear. One possible explanation is that the body mass index is not capable of precisely quantifying body fat.

The results found have significant practical applications. Knowing that central obesity is inversely related to the parasympathetic modulation in the heart of obese normotensive adolescents, one can consider such a relationship an early marker of cardiovascular disturbances in this population, which may result in cardiovascular diseases. This fact makes the issue clinically relevant, since it is known^(28,29)^ that cardiovascular diseases originated during the initial phases of life (childhood and adolescence) remain in adult life. Thus, the identification of adolescents with greater cardiovascular risk may provide information as to the individuals who need treatment.

The present study has some limitations that should be considered. The cross-sectional design precludes the establishment of causality among the dependent and independent variables. The use of double X-ray absorptiometry for the analysis of body composition enables corporal segmentation as to the sites of accumulated adipose tissue. Additionally, no blood was collected, which would allow dosing of inflammatory markers, hormones, and adipokines, thus enabling understanding of the mechanisms involved in the results found^(30–32)^.

## CONCLUSION

Central obesity is a better discriminator of autonomic cardiac dysfunction in obese normotensive adolescents than general obesity.
